# Adaptation and measurement invariance of the 13-item version of Patient Activation Measure across Japanese young adult cancer survivors during and after treatment: A cross-sectional observational study

**DOI:** 10.1371/journal.pone.0291821

**Published:** 2023-09-19

**Authors:** Takafumi Soejima, Mari Kitao

**Affiliations:** Department of Child Health Nursing, Graduate School of Health Sciences, Kobe University, Kobe, Hyogo, Japan; Sunway University, MALAYSIA

## Abstract

The 13-item version of the Patient Activation Measure (PAM-13) is a frequently used measure that gauges the level of self-management in an individual. However, its applicability across Japanese young adult (YA) cancer survivors during and after their treatment remains unclear. This study confirmed the psychometric properties and measurement invariance of the Japanese version of PAM-13 across them during and after treatment. We used cross-sectional observational data collected through an online survey from 500 survivors in January 2022. We determined feasibility, internal consistency, concurrent validity against physical fatigue and depression, and known-groups validity regarding educational level. Structural validity was also found using Rasch analysis for survivors both during and after treatment. Furthermore, measurement invariance of the PAM-13 was examined using multiple-group structural equation modeling. Rasch fit statistics were acceptable for the unidimensional structure of PAM-13. It was found to be internally consistent for survivors during (McDonald’s omega: 0.88, item-total correlations: 0.48–0.62) and after treatment (McDonald’s omega: 0.90, item-total correlations: 0.32–0.72). The PAM-13 was concurrently valid with physical fatigue (Pearson’s product-moment correlation coefficients: -0.25 and -0.18 for survivors during and after treatment, respectively) and depression (Pearson’s product-moment correlation coefficients: -0.20 and -0.19 for survivors during and after treatment, respectively). Known-groups validity showed that survivors after treatment with a higher educational level reported a higher patient activation score than those with a lower educational level (*p* = 0.001); however, there was no difference due to the education level between survivors during treatment. The configural and metric invariance of the PAM-13 were confirmed, but scalar invariance was rejected. It was found that the PAM-13 is applicable for Japanese YA cancer survivors during and after treatment. However, given the lack of scalar invariance in the PAM-13, the scores of particular items between YA cancer survivors during and after treatment should be interpreted with caution.

## Introduction

Individuals in young adulthood typically separate from their parents, begin romantic relationships that may lead to marriage, pregnancy, and childbirth, and also develop their careers of education and employment, which may coincide with the establishment of their identities [1]. However, it becomes difficult for young adult (YA) cancer survivors to undergo these life stages as they battle with the symptoms of the disease [2–4]. Thus, handling cancer-related symptoms is essential for YA cancer survivors to overcome these challenges and improve their health-related quality of life [5, 6].

The Chronic Care Model suggests that a productive interaction between an activated patient with skills, knowledge, and motivation and a care team is essential to improve the patient’s health outcomes [7]. On the basis of this model, Hibbard et al. defined patient activation as an individual’s knowledge, skills, and confidence in managing their own health [8]. Studies have focused on patient activation and have noted that it plays an important role in improving health outcomes among individuals with chronic conditions, including cancer [9, 10]. The 13-item version of the Patient Activation Measure (PAM-13) is one of the most frequently used measures worldwide to assess patient activation in individuals with chronic conditions [11]. Existing studies have confirmed the original and other language versions of PAM-13 as a unidimensional scale that indicates the degree of activation that helps chronically ill individuals become fully engaged in managing their health [11–13]. Furthermore, the studies validated the scale for application in individuals with various chronic conditions (e.g., arthritis, hypertension, chronic pain, kidney disease, and diabetes) [11, 14–16] and for the general population [12]. Previous studies commonly reported that among the participants, demographic characteristics (e.g., older age and lower educational level), health condition (e.g., depression, fatigue, and poor quality of life), and lower self-efficacy on controlling for disease and symptom were negatively related to the score of PAM-13 [17–20]. The Japanese version of the PAM-13 was developed to confirm the following reliability and validity: internal consistency using Cronbach’s alpha, test-retest reliability, unidimensionality using Rasch analysis, and concurrent validity against self-efficacy for adherence to treatment [21].

Despite the fact that cancer presents various disease-related complexities of disease, risks of treatment and late effect, and longer-term disability than many other chronic conditions, cancer care has lagged behind other chronic conditions in incorporating self-management into routine care [22]. Treatment and follow-up after treatment are performed as an outpatient rather than as an inpatient since chemotherapy and radiotherapy are proved to be safe enough to require less intensive monitoring by healthcare professionals [23]. Thus, healthcare staff should encourage cancer survivors both during and after treatment to participate in their self-care and manage treatment-related symptoms, and evaluate their capacity and capability to self-manage their symptoms [23, 24].

Cancer survivors experience a range of physical, psychological, and cognitive problems during and after their cancer treatment. More than half of adolescent and YA cancer survivors receiving myelosuppressive chemotherapy reported a lack of energy, feeling drowsy, difficulty sleeping, and pain as the most prominent symptoms. They managed their symptoms with medication and physical care like getting proper nutrition and physical exercise [25]. Furthermore, even after completing their treatment, cancer survivors including young adults report chronic fatigue, depression, and cognitive impairment [26–28]. YA cancer survivors often use psychosocial strategies such as managing their emotions and seeking support from others [29]. After treatment, they require regularly participating in routine, but less frequently than those during treatment [23]. Thus, often with minimal clinical advice or supervision, they need to monitor the signs and symptoms of cancer recurrence, adjust to the late-term effects of treatment, re-establish social roles, and deal with psychological distress, to minimize negative impacts on quality of life [23]. However, they often have to take on more responsibility for the day-to-day management while lacking confidence for initiating the management necessary to recover health after treatment [22].

Although PAM-13 was used to assess cancer survivors’ patient activation [30, 31], the patient activation characteristics may differ between cancer survivors during and after treatment because of factors like varied range of symptoms, involvement with healthcare staff, and responsibility for self-management during and after treatment. However, no study has clarified whether PAM-13 was equally applicable in cancer survivors during and after treatment. This study aimed to examine the psychometric properties and measurement invariance of the Japanese version of the PAM-13 in YA cancer survivors both during and after treatment. Our findings may help provide insights into the applicability and interpretation of patient activation across YA cancer survivors during and after treatment.

## Materials and methods

### Study participants and procedure

This study used data from a cross-sectional observational study as part of which participants completed an online survey comprising questionnaires designed to assess work sustainability and cancer-related symptoms (physical fatigue and depression). We recruited YA cancer survivors in January 2022 through the commercial panel system of Macromill, Inc. (https://group.macromill.com), an Internet research company in Japan. Inclusion criteria of this study were: participants had to be 20–39 years of age, diagnosed with cancer at the age of 20 years or older, and could understand the purpose of this study and complete a questionnaire in Japanese.

The online questionnaire was administered via e-mail through Macromill, Inc. to all 6,161 cancer survivors aged 20–39 years who had registered spontaneously with Macromill, Inc. The e-mail contained the link to the online questionnaire system provided by Macromill, Inc. The participants logged into the online questionnaire system on their own devices (tablets, smartphones, etc.) and were then informed about this study on the website. The participants could answer the questionnaire only if they consented to participate in this study. They were compensated for their participation in the form of points according to the number of questions answered; these points could then be redeemed as cash or exchanged for items.

Of the 6,161 participants, 693 returned the online questionnaires (response rate, 11.2%). This response rate was similar to the previous study [32]. Of the 693 participants who returned the online questionnaire, 138, who were diagnosed with cancer when they were younger than 20, and 55 whose treatment status at the time of the survey was unknown, were excluded. Finally, data from a total of 500 YA cancer survivors were analyzed for this study.

### Ethical consideration

This study was performed in accordance with the principles of the Declaration of Helsinki and Ethical Guidelines for Medical and Biological Research Involving Human Subjects established by the Ministry of Health, Labor, and Welfare. This study was approved by the Ethics Committee of the principal investigator’s institution (August 31, 2021, No. 1029). Before participating in this study, the participants were informed that they were free to choose to participate in this study and would not face any disadvantage if they did not participate in the study. After being informed about the study, the participants provided consent to participating in our study by answering “yes” to the question “Do you consent to participate in this study?” on the survey platform provided by Macromill, Inc. Their responses regarding participation in our study were digitally recorded. The participants had to consent before they could access the questionnaire. The responses to the questionnaire were recorded anonymously.

### Measures

Patient activation was assessed using the Japanese version of the PAM-13, a unidimensional scale, which includes 13 items on respondents’ knowledge, skills, and confidence in managing their health (S1 Table) [11, 21]. The responses of each item were rated on 4-point Likert scales, ranging from “Disagree Strongly (1)” to “Agree Strongly (4)” and “Not Applicable.” The option “Not Applicable” was computed as a missing value. The patient activation score was calculated from the raw score based on an empirically derived calibration table and ranged from 0 to 100. A higher patient activation score indicates more proactive involvement in management of their own health. If more than half of the items were missing, the patient activation score was excluded.

The Physical Fatigue subscale of the Cancer Fatigue Scale (CFS) was used to measure physical fatigue in this study [33]. This scale comprises seven items that are to be scored using a five-point Likert scale. The score was calculated as the sum of the scores of all items. If more than half of the items had missing responses, the score was not calculated. The possible subscale scores range from 0 to 28, with a higher score indicating more severe physical fatigue. The Cronbach’s alpha in this study was 0.94.

The Kessler Psychological Distress Scale (K6) was used to measure depressive symptoms among the study participants [34]. It includes six items whose responses are rated on a 5-point Likert scale. The K6 score was calculated as the sum of the scores for all the items. If more than half of the items had missing responses, the K6 score was not computed. The score ranges from 0 to 24, with higher scores indicating more severe depressive symptoms. The Cronbach’s alpha in this study was 0.94.

Furthermore, the participants were asked to record their age at the time of the survey, gender, educational level, cancer site, the period from diagnosis, treatment status, treatment modalities, metastasis, and relapse. We categorized participants who were receiving cancer treatment at the time of the survey into the during-treatment group and those who completed cancer treatment at the time of the survey into the after-treatment group, based on the answer to the question about treatment status.

### Statistical analyses

All analyses were performed using IBM SPSS software version 24 and Amos software version 28 (SPSS, Inc., Chicago, IL, USA). The level of significance was set at *p* < 0.05.

#### Demographic and clinical characteristics

Frequencies, percentages, means, or standard deviations (SD) of demographic and clinical characteristics were calculated, and these variables were compared between the during- and after-treatment groups using the chi-squared test and Welch’s t-test. Furthermore, Cohen’s omega (≥ 0.1, small; 0.3, medium; 0.5, large) for categorical variables and Cohen’s d (≥ 0.2, small; 0.5, medium; 0.8, large) for continuous variables were calculated as effect sizes [35].

#### Score distribution

Score distributions for the PAM-13 were summarized as mean, standard deviation (SD), range, and percentages of floor and ceiling scores by treatment status. A ceiling or floor effect was defined as 15% or more of the individuals in a sample achieving the best or worst score [36]. Thereafter, we compared the mean scores between the during- and after-treatment groups using Welch’s t-test, and calculated Cohen’s d (≥ 0.2, small; 0.5, medium; 0.8, large) as effect sizes [35].

#### Feasibility

Feasibility was determined based on the percentage of missing values and independence of missed items assessed using Cochran’s Q test. For this study, we estimated the percentage of missing values and conducted Cochran’s Q test for the during- and after-treatment groups.

#### Construct validity

We hypothesized that the PAM-13 was a one-factor structure on the basis of the original and other language versions of PAM-13 [11–13]. Rasch rating scale analysis was performed to confirm the unidimensional structure of PAM-13 using data of 448 participants who answered all items of the scale. We estimated the information-weighted fit (Infit) statistics and outliner-sensitive fit (Outfit) statistics of the items, and eigenvalues in the principal component analysis of standardized residuals for the during- and after-treatment groups. Infit and Outfit statistics between 0.5 and 1.5, and a first component’s eigenvalue less than 2.0 are an acceptable fit to the unidimensional model [37, 38]. Furthermore, we estimated item locations that represent the degree to which respondents have difficulty agreeing with the content of the items. A higher item location indicated that an item is more difficult for respondents to agree with.

#### Reliability

Reliability was assessed based on internal consistency for both groups. Good internal consistency was defined as a McDonald’s omega value exceeding 0.70 [39] and item-total correlation (Pearson’s product-moment correlation coefficients) exceeding 0.30 [40].

#### Concurrent, known-group, and convergent validity

The concurrent, known-group, and convergent validity of PAM-13 were assessed in both the groups. Concurrent validity was checked by calculating Pearson’s product-moment correlation coefficients and it was confirmed that the patient activation score was negatively correlated with the CFS Physical Fatigue subscale and K6 scores because high patient activation was associated with better self-rated health [17–20]. The patient activation scores of YA cancer survivors with higher (graduate from college, vocational school, university, and graduate school) and lower educational levels (graduate from junior high school, high school, and others) were compared using Welch’s t-test to describe known-group validity. Furthermore, we calculated Cohen’s d as effect sizes. We also predicted that the patient activation score would be low among YA cancer survivors with lower educational levels based on the previous studies of the PAM-13 [17–20]. Previous studies reported the association between age and patient activation. However, we did not confirm differences in the patient activation score by age in this study because our study participants were young. Convergent validity was checked by calculating average variance extracted (AVE) and composite reliability (CR) [41]. Adequate convergent validity was defined as AVE exceeding 0.50 and CR exceeding 0.60 [41]. Interestingly, even if AVE is less than 0.50, but composite reliability is higher than 0.60, the convergent validity of the construct is still adequate [41].

#### Measurement invariance

Measurement invariance of PAM-13 between the two groups was assessed using multiple-group structural equation modeling (SEM) [42] using the data of 448 participants who answered all items of the PAM-13. First, we tested the configural invariance, which represents the same factor structure across the groups, based on goodness-of-fit. An acceptable fit was indicated by the chi-square statistic divided by degrees of freedom (CMIN/df) < 3, comparative fit index (CFI) > 0.95, goodness-of-fit index (GFI) > 0.90, adjusted goodness-of-fit index (AGFI) > 0.85, root mean square error of approximation (RMSEA) < 0.08, and standardized root mean square residual (SRMR) < 0.10 [43]. Second, we sequentially applied the equality constraints of factor loadings and intercepts of the observed variables between the two groups [42]. The application of more equality constraints led to a poorer model fit. For testing invariance of factor loadings, it was noted that a change of -0.010 in CFI, supplemented by a change of 0.015 in RMSEA or a change of 0.030 in SRMR did not indicate invariance; for testing invariance of intercept of observed variables, a change of -0.010 in CFI, supplemented by a change of 0.015 in RMSEA or a change of 0.010 in SRMR did not indicate invariance [42]. If the equality constraint of factor loadings was applicable under configural invariance, the metric invariance between the two groups was confirmed. Furthermore, if the equality constraint of the intercepts of the observed variables was applicable under metric invariance, scalar invariance was confirmed. However, if metric and scalar invariances were not confirmed, we tested which factor loadings or intercepts of the observed variables differed using z-statistics. We evaluated the fit of the model without constraining factor loadings or intercepts of the observed variables that differed between the groups. Configural, metric, and scalar invariances are necessary to determine whether the two groups are psychometrically equivalent [44].

## Results

### Sample characteristics

Of the 500 participants whose data were analyzed, 237 (47%) were in the during-treatment group and 263 (53%) in the after-treatment group (Table 1). Approximately half of the participants (n = 246, 49%) were aged 35–39 years, 349 (70%) were female, and 276 (55%) had graduated from university or graduate school. The most common diagnoses were uterine and ovarian cancer (n = 147, 29%), and more than half of the participants underwent surgery (n = 323, 65%). Participants’ characteristics excluding surgery significantly differed between the on- and after-treatment groups.

**Table 1:** Demographic and clinical characteristics of the participants.

N = 500									
	Total		During-treatment group (n = 237)		After-treatment group (n = 263)				
	n	%	n	%	n	%	χ^2 a^	*p* ^a^	ω ^a^
Age at the time of the survey (in years)							13.29	0.004	0.16
20–24	22	4	13	5	9	3			
25–29	80	16	51	22	29	11			
30–34	152	30	71	30	81	31			
35–39	246	49	102	43	144	55			
Gender							47.76	< 0.001	0.31
Male	151	30	107	45	44	17			
Female	349	70	130	55	219	83			
Educational level							20.08	0.001	0.20
Junior-high school	13	3	5	2	8	3			
High school	101	20	39	16	62	24			
College‎/Vocational school	100	20	35	15	65	25			
University‎/Graduate school	276	55	151	64	125	48			
Others	10	2	7	3	3	1			
Cancer site							92.97	< 0.001	0.43
Gastric cancer	64	13	48	20	16	6			
Colorectal cancer	47	9	32	14	15	6			
Breast cancer	73	15	48	20	25	10			
Uterine and ovarian cancer	147	29	27	11	120	46			
Thyroid cancer	33	7	12	5	21	8			
Lymphoma‎/Leukemia	32	6	12	5	20	8			
Others ^b^	104	21	58	24	46	17			
Period from diagnosis							66.00	< 0.001	0.36
< 1 years	129	26	95	40	34	13			
1 - < 5 years	239	48	111	47	128	49			
≥ 5 years	132	26	31	13	101	38			
Treatment modalities									
Chemotherapy	227	45	163	69	64	24	66.60	< 0.001	0.37
Surgery	323	65	158	67	165	63	0.84	0.359	0.04
Radiation	156	31	121	51	35	13	82.75	< 0.001	0.41
Hormone therapy	120	24	100	42	20	8	81.77	< 0.001	0.40
HCT	37	7	33	14	4	2	27.99	< 0.001	0.24
Metastasis	81	16	76	32	5	2	83.56	<0.001	0.41
Relapse	106	21	94	40	12	5	91.94	< 0.001	0.43
	Mean	SD	Mean	SD	Mean	SD	t ^a^	p ^a^	d ^a^
CFS Physical Fatigue	11.5	7.9	14.2	7.3	9.1	7.6	7.62	< 0.001	0.68
K6	8.8	7	11.4	6.7	6.4	6.5	8.53	< 0.001	0.77

Note. CFS, Cancer Fatigue Scale; K6, Kessler 6; HCT, Hematopoietic cell transplantation. ^a^ The *p*-values using chi-square test and Cohen’s ω were calculated for categorical variables, and the *p*-values using Welch’s t-test and Cohen’s d were calculated for continuous variables. ^b^ Lung cancer (N = 19), liver cancer (N = 17), oral cancer (N = 15), pancreatic cancer (N = 6), prostate cancer (N = 5), renal cancer (N = 5), cancer of central nervous system (N = 2), and others (N = 35).

### Score distribution

The values for the items of the PAM-13 represented a possible range of 1–4 (Table 2). The patient activation score was 9.0–100.0 in the during-treatment group and 17.9–100.0 in the after-treatment group. Although no floor effect was observed, ceiling effects were observed for all items in the during-treatment group and for all items excluding item 13 in the after-treatment group. However, only 3% of participants in both the on- and after-treatment groups reported the maximum possible patient activation score. Participants in the after-treatment group reported higher scores on items 1 (*p* < 0.001) and 2 (*p* < 0.001), and those in the during-treatment group reported higher scores on items 10 (*p* = 0.016), 12 (*p* = 0.016), and 13 (*p* = 0.001). The mean patient activation score was 55.5 (SD 15.4) in the during-treatment group and 55.3 (SD 15.6) in the after-treatment group, which reflected no significant difference (*p* = 0.420).

**Table 2:** Item score and item-total correlation of the PAM-13.

N = 500																	
	During-treatment group (n = 237)							After-treatment group (n = 263)									
	n	Mean	SD	Range	Floor	Ceiling	r ^a^	n	Mean	SD	Range	Floor	Ceiling	r ^a^	t ^b^	*p* ^b^	d ^b^
Item 1	229	3.1	1.0	1.0–4.0	8%	44%	0.51	263	3.5	0.7	1.0–4.0	2%	57%	0.32	-4.77	< 0.001	0.44
Item 2	232	3.0	0.8	1.0–4.0	4%	25%	0.57	261	3.2	0.7	1.0–4.0	2%	37%	0.45	-3.76	< 0.001	0.34
Item 3	233	2.8	0.8	1.0–4.0	6%	22%	0.60	262	2.8	0.8	1.0–4.0	4%	18%	0.66	0.82	0.205	0.07
Item 4	226	3.0	0.8	1.0–4.0	4%	31%	0.59	258	3.0	0.7	1.0–4.0	3%	24%	0.51	0.50	0.310	0.05
Item 5	234	2.9	0.9	1.0–4.0	5%	29%	0.61	259	2.8	0.8	1.0–4.0	4%	18%	0.70	1.03	0.151	0.09
Item 6	231	2.9	0.8	1.0–4.0	6%	24%	0.56	261	3.0	0.8	1.0–4.0	5%	27%	0.63	-0.54	0.295	0.05
Item 7	231	2.9	0.8	1.0–4.0	4%	24%	0.55	262	3.0	0.8	1.0–4.0	4%	28%	0.57	-0.96	0.168	0.09
Item 8	230	3.0	0.8	1.0–4.0	3%	28%	0.55	263	3.0	0.7	1.0–4.0	2%	22%	0.63	0.93	0.176	0.08
Item 9	229	2.9	0.8	1.0–4.0	4%	24%	0.62	259	2.9	0.8	1.0–4.0	4%	23%	0.67	0.06	0.477	0.01
Item 10	231	2.9	0.8	1.0–4.0	5%	26%	0.52	261	2.7	0.9	1.0–4.0	9%	20%	0.64	2.16	0.016	0.19
Item 11	232	2.8	0.8	1.0–4.0	5%	21%	0.55	260	2.8	0.8	1.0–4.0	5%	17%	0.72	0.86	0.196	0.08
Item 12	235	2.8	0.8	1.0–4.0	4%	23%	0.55	259	2.7	0.9	1.0–4.0	8%	19%	0.72	2.15	0.016	0.19
Item 13	233	2.8	0.8	1.0–4.0	5%	17%	0.48	258	2.5	0.9	1.0–4.0	14%	14%	0.54	3.19	0.001	0.29
Patient activation score	237	55.5	15.4	9.0–100.0	0%	3%	N/A	263	55.3	15.6	17.9–100.0	0%	3%	N/A	0.20	0.420	0.02

Note. N/A, Not applicable; SD, Standard deviation. ^a^ Item-total correlation. ^b^ The *p*-values using Welch’s t-test and Cohen’s d were calculated.

### Feasibility

The average percentage of missing responses was found to be 1.7% (2.4% in the during-treatment group and 1.0% in the after-treatment group). Missing items were independent of each other in both the during-treatment (*p* = 0.264) and after-treatment (*p* = 0.154) groups.

### Construct validity

In the Rasch analysis, the infit statistics were 0.73–1.40 in the during-treatment group and 0.67–1.47 in the after-treatment group (Table 3). The Outfit statistics were 0.72–1.30 in the during-treatment group and 0.68–1.38 in the after-treatment group. The eigenvalues of the first component in a principal component analysis of standardized residuals were 1.76 and 1.92 for the during-treatment and after-treatment groups, respectively. Item locations tended to progressively increase from items 1 to 13 in both groups.

**Table 3:** Item location and infit/outfit value of the PAM-13.

N = 448						
	During-treatment group (n = 204)			After-treatment group (n = 244)		
	Item location	Infit	Outfit	Item location	Infit	Outfit
Item 1	1.48	1.40	1.30	0.40	1.47	1.38
Item 2	1.86	0.87	0.85	1.28	1.19	1.16
Item 3	2.24	0.91	0.96	2.57	0.77	0.78
Item 4	1.73	0.94	0.97	1.91	1.02	1.08
Item 5	2.06	0.92	0.89	2.38	0.68	0.67
Item 6	1.97	0.91	0.93	1.99	0.94	0.92
Item 7	1.99	0.87	0.86	1.94	1.01	1.06
Item 8	1.75	0.83	0.85	2.03	0.73	0.73
Item 9	1.97	0.73	0.72	2.12	0.75	0.75
Item 10	2.12	1.02	0.98	2.73	1.00	1.02
Item 11	2.28	0.93	0.93	2.55	0.67	0.68
Item 12	2.20	0.91	0.89	2.80	0.78	0.77
Item 13	2.42	0.96	1.06	3.20	1.19	1.25

Note. Infit, Information-weighted fit; Outfit, Outlier-sensitive fit.

### Reliability

McDonald’s omega were 0.88 in the during-treatment group and 0.90 in the after-treatment group. Item-total correlations were 0.48–0.62 in the during-treatment and 0.32–0.72 in the after-treatment group (Table 2).

### Concurrent, known-group, and convergent validity

The patient activation score was found to be negatively associated with the Physical Fatigue subscale score of the CFS (r = -0.25 in the during-treatment group and r = -0.18 in the after-treatment group) and K6 score (r = -0.20 in the during-treatment group and r = -0.19 in the after-treatment group) in both the groups (Table 4). Validation for known-groups showed that participants with a high educational level reported a higher patient activation score than those with a low educational level (*p* = 0.001) in the after-treatment group (Table 5). However, there was no significant difference in the during-treatment group (*p* = 0.594). Regarding convergent validity, the AVE and CR were 0.34 and 0.86 for the during-treatment, and 0.42 and 0.90 for the after-treatment group, respectively.

**Table 4:** Concurrent validity of the PAM-13.

N = 500				
	CFS Physical Fatigue		K6	
	r ^a^	*p* ^a^	r ^a^	*p* ^a^
During-treatment group (n = 237)				
Patient activation score	-0.25	<0.001	-0.20	0.002
After-treatment group (n = 263)				
Patient activation score	-0.18	0.003	-0.19	0.002

Note. CFS, Cancer Fatigue Scale; K6, Kessler 6. ^a^ Correlation coefficients and these *p*-values were calculated by Pearson’s product moment correlation analysis.

**Table 5:** Known-groups validity of the PAM-13.

N = 500									
	High educational level ^a^			Low educational level ^a^					
	n	Mean	SD	n	Mean	SD	t ^b^	*p* ^b^	d ^b^
During-treatment group (n = 237)									
Patient activation score	186	55.2	15.1	51	56.6	16.3	-0.54	0.594	0.09
After-treatment group (n = 263)									
Patient activation score	190	57.1	15.9	73	50.5	13.5	3.34	0.001	0.43

Note. SD, Standard deviation. ^a^ High educational level included participants who graduated from college, vocational school, university, and graduate school, and low educational level included participants who graduated from other schools. ^b^ The *p*-values using Welch’s t-test and Cohen’s d were calculated.

### Measurement invariance

Multiple-group SEM with the same structure showed an acceptable fit (CMIN/df = 1.80, CFI = 0.954, GFI = 0.929, AGFI = 0.894, RMSEA = 0.042, and SRMR = 0.049), thereby confirming configural invariance. Moreover, the model that applied equality constraints of factor loadings was acceptable (ΔCFI = -0.009, ΔRMSEA = 0.003, and ΔSRMR = 0.012), thereby confirming metric invariance (Table 6). However, the model that applied the equality constraints of intercepts of observational variables was not acceptable (ΔCFI = -0.030, ΔRMSEA = 0.008, and ΔSRMR = 0.018), and scalar invariance was rejected. The test using z-statistics also indicated significant differences between the two groups regarding items 1 (Z = 4.59, *p* < 0.001), 2 (Z = 3.36, *p* < 0.001), 10 (Z = 2.14, *p* = 0.016), 12 (Z = 2.09, *p* = 0.018), and 13 (Z = 2.87, *p* = 0.002). The model that did not apply the equality constraints of intercepts of observational variables for items 1, 2, 10, 12, and 13 had a better fit than the fit of the model with constraining all intercepts of the observed variables (ΔCFI = -0.009, ΔRMSEA = 0.001, and ΔSRMR = 0.012).

**Table 6:** Model fit of PAM-13 between the during-treatment and after-treatment groups.

N = 448											
	CMIN	*p* for CMIN	CMIN/df	CFI	GFI	AGFI	RMSEA	SRMR	ΔCFI	ΔRMSEA	ΔSRMR
Configural invariance	219.59	<0.01	1.80	0.954	0.929	0.894	0.042	0.049	N/A	N/A	N/A
Metric invariance	255.21	<0.01	1.91	0.945	0.917	0.887	0.045	0.061	-0.009	0.003	0.012
Scalar invariance	308.84	<0.01	2.10	0.924	N/A ^a^	N/A ^a^	0.050	0.067	-0.030	0.008	0.018
Partial scalar invariance ^b^	260.39	<0.01	1.83	0.945	N/A ^a^	N/A ^a^	0.043	0.061	-0.009	0.001	0.012

Note. CFI, Comparative Fit Index; CMIN, Chi-square values; df, Degree of freedom; N/A, Not applicable; RMSEA, Root Mean Square Error of Approximation; SRMR, Standardized Root Mean square Residual. ^a^ The GFI and AGFI were not able to be calculated because the mean and intercept of observational variables are estimated in Amos software. ^b^ The model did not apply the equality constraints of intercepts of observational variables for items 1, 2, 10, 12, and 13.

## Discussion

Our study demonstrated that the feasibility, reliability, and validity of the Japanese version of the PAM-13 were confirmed across YA cancer survivors during and after treatment through multiple statistical methods, excluding known-group validity in the participants during treatment. The results suggest applicability of the Japanese version of the PAM-13 for YA cancer survivors during and after treatment. Moreover, the multiple-group SEM supported configural and metric invariance between YA cancer survivors during and after treatment. However, we did not confirm its scalar invariance.

Similarly, this study found that almost all items on the PAM-13 had a ceiling effect, which corresponded to previous studies of adolescents with chronic conditions and kidney disease [14, 15]. However, in the study, only 3% of the participants in both groups reported the maximum possible patient activation score. Thus, it was found that the ceiling effects in the items of the PAM-13 would not influence the evaluation of patient activation for YA cancer survivors using the PAM-13. The feasibility of the PAM-13 was found to be high across YA cancer survivors during and after treatment based on the proportion and independence of the missing items.

The Rasch analysis results and the multiple-group SEM with the same configuration suggest that the PAM-13 is a single underlying factor across YA cancer survivors during and after treatment. The unidimensional structure of the scale has been observed in the original study by Hibbard et al. [11] that dealt with the original version and other studies that dealt with other language versions [12, 13]. The results of McDonald’s omegas and item-total correlations supported the good internal consistency of the PAM-13 across YA cancer survivors during and after treatment.

Our study’s findings support the concurrent validity of patient activation against physical fatigue and depression. Known-group validity was confirmed for YA cancer survivors after treatment, but not among them during treatment. Among patients with chronic conditions, a higher educational level translated into better knowledge, attitudes, and skills related to the management and control of their diseases, which implied that individuals most easily put their obtained knowledge and skills into practice for self-management [45–47]. However, YA cancer survivors during treatment responded better to taking medications rather than individual coping strategies, like making healthy lifestyle changes [24]. Thus, even if YA cancer survivors have a higher educational level, they may depend on pharmacological therapy and play a passive role in handling their side-effects during treatment. This could be the reason that known-group validity was not confirmed in the during-treatment group.

In this study, the fit of the model with the equality constraints of factor structure and factor loadings between YA cancer survivors during and after treatment was acceptable; but the fit of the model with the equality constraints of intercept of observational variables was not poor. Furthermore, the fit of the model that did not apply the equality constraints of intercepts of observational variables that differed between YA cancer survivors during and after treatment (items 1, 2, 10, 12, and 13) was better than the fit of the model with constraining all intercepts of the observed variables. Our findings supported configural and metric invariance of the PAM-13 and rejected scalar invariance. This implies that the scores of items 1, 2, 10, 12, and 13 do not necessarily mean the same thing for YA cancer survivors during and after treatment. Thus, the patient activation score could not be compared directly between YA cancer survivors during and after treatment.

Our study found that YA cancer survivors after treatment reported higher scores on items 1 and 2, indicating a belief in an active role in self-management. One possible reason for this could be that cancer survivors after treatment are less likely to receive clinical advice or supervision and need to handle cancer-related symptoms and manage their care independently [23]. The YA cancer survivors after treatment took greater responsibility for self-managing their recovery [48], which represented a higher score for Items 1 and 2 in them after treatment.

Contrarily, YA cancer survivors during treatment reported higher scores on items 10, 12, and 13, which indicate taking and continuing actions for self-management. In a previous study, YA cancer survivors who received myelosuppressive chemotherapy often engaged in the management of lifestyle habits (e.g., eating and physical activity), which were shown in Items 10 and 13, and shared this management with their healthcare team [24]. Cancer survivors undergoing treatment also have more opportunities to visit hospitals for treatment and receive advice and support for self-management from healthcare staff [23]. The healthcare staff communicated and provided developmentally appropriate care for YA cancer survivors during chemotherapy to understand their symptom experiences and proceed with self-management efforts [24]. This kind of support from the healthcare staff would enable YA cancer survivors to take and continue their actions for self-management. Furthermore, individuals in young adulthood possibly engage more in self-management due to greater social expectations for them to rise to the occasion and face adversity [24]. Thus, YA cancer survivors during treatment may implement and sustain self-management practices in response to expectations from the healthcare staff who support them.

In our study, there was no significant difference in the scores of items 3–9, indicating confidence and knowledge for self-management, between YA cancer survivors during and after treatment. Since three-quarters of total YA cancer survivors were diagnosed more than a year after diagnosis, it is possible that they had already obtained sufficient knowledge and confidence regarding self-management. YA cancer survivors desire to be part of the treatment process and value an alliance with their healthcare staff [49–51]. Therefore, our study participants both during and after treatment may have a good relationship with their healthcare staff and have confidence regarding communication with their healthcare staff indicated by items 5, 6, and 7. Furthermore, YA cancer survivors have the desire to learn about self-management options and skills [48, 52]. In our study, many YA cancer survivors had been diagnosed more than a year ago, and they had already obtained sufficient knowledge regarding cancer and its therapy, as indicated in items 4, 8, and 9, regardless of whether during or after treatment.

We consider the advantage of this study to be to firstly assess and confirm the feasibility, reliability, and validity for each of the YA cancer survivors during and after treatment, and to test a sample with a variety of cancers diagnosed in young adulthood. However, our study has several limitations. Most of our study participants were women, reflecting the high proportion of female cancer survivors aged 20–39 years in Japan [53]. Furthermore, the participants had a higher educational level, considering that half of the participants graduated from university or graduate school [54]. Studies have reported that educational level is positively related to activation in patients with chronic diseases [17–20]. Our study may have overestimated the item scores and patient activation scores of the PAM-13 among YA cancer survivors. Therefore, further validation of the Japanese version of the PAM-13 is needed for YA cancer survivors from diverse socio-demographic backgrounds, including male survivors and survivors with a lower educational level. Furthermore, the PAM-13 contains items common to self-management in individuals with a variety of chronic conditions. The development of a scale measuring self-management knowledge, skills, and motivation specific to young adult cancer survivors would more appropriately measure their patient activation.

## Conclusion

The Japanese version of the PAM-13 was generally suitable across YA cancer survivors during and after treatment to assess their patient activation because the validity and reliability of the PAM-13 were confirmed excluding known-groups validity in YA cancer survivors during treatment. However, we did not confirm scalar invariance of the PAM-13 between YA cancer survivors during and after treatment. Thus, the patient activation score of the PAM-13 could not be compared directly between YA cancer survivors during and after treatment. Given the limitations of the present study, our findings require further validation of the PAM-13 for male YA cancer survivors and YA cancer survivors with low level of education.

## Supporting information

S1 Checklist STROBE statement—checklist of items that should be included in reports of observational studies. (DOCX)

S1 Table Items on the PAM-13. (DOCX)
