# Supplementary material for: Adaptation and measurement invariance of the 13-item version of Patient Activation Measure across Japanese young adult cancer survivors during and after treatment: A cross-sectional observational study
Source: PLoS One. 2023 Sep 19;18(9):e0291821. doi: 10.1371/journal.pone.0291821 (PMC10508623; doi:10.1371/journal.pone.0291821)
Supplement: S1 Checklist — (DOCX) [file pone.0291821.s001.docx]

STROBE Statement—checklist of items that should be included in reports of observational studies

|  | Item No. | Recommendation | Page  No. | Relevant text from manuscript |
| --- | --- | --- | --- | --- |
| **Title and abstract** | 1 | (*a*) Indicate the study’s design with a commonly used term in the title or the abstract | 1 | Title: Adaptation and measurement invariance of the 13-item version of Patient Activation Measure across Japanese young adult cancer survivors during and after treatment: A cross-sectional observational study  Abstract: We used cross-sectional observational data… |
|  |  | (*b*) Provide in the abstract an informative and balanced summary of what was done and what was found | 2 | Please see the abstract. |
| Introduction | | | |  |
| Background/rationale | 2 | Explain the scientific background and rationale for the investigation being reported | 3-6 | Please see the Introduction. |
| Objectives | 3 | State specific objectives, including any prespecified hypotheses | 6 | Thus, this study aimed to examine the psychometric properties and measurement invariance of the Japanese version of the PAM-13 in YA cancer survivors both during and after treatment. |
| Methods | | | |  |
| Study design | 4 | Present key elements of study design early in the paper | 6 | This study used data obtained from a cross-sectional observational study… |
| Setting | 5 | Describe the setting, locations, and relevant dates, including periods of recruitment, exposure, follow-up, and data collection | 5-6 | Please see the ‘Study participants and procedure’ in the Methods and materials. |
| Participants | 6 | (*a*) *Cohort study*—Give the eligibility criteria, and the sources and methods of selection of participants. Describe methods of follow-up  *Case-control study*—Give the eligibility criteria, and the sources and methods of case ascertainment and control selection. Give the rationale for the choice of cases and controls  *Cross-sectional study*—Give the eligibility criteria, and the sources and methods of selection of participants | 5-6 | Inclusion criteria of this study were: participants had to be 20–39 years of age, diagnosed with cancer at age 20 years or older, and could understand the purpose of this study and complete a questionnaire in Japanese. |
|  |  | (*b*) *Cohort study*—For matched studies, give matching criteria and number of exposed and unexposed  *Case-control study*—For matched studies, give matching criteria and the number of controls per case | N/A |  |
| Variables | 7 | Clearly define all outcomes, exposures, predictors, potential confounders, and effect modifiers. Give diagnostic criteria, if applicable | 8-9 | Please see the ‘Measures’ in the Methods and materials. |
| Data sources/ measurement | 8* | For each variable of interest, give sources of data and details of methods of assessment (measurement). Describe comparability of assessment methods if there is more than one group | 8-9 | Please see the ‘Measures’ in the Methods. We categorized participants who were receiving cancer treatment at the time of the survey into the on-treatment group and those who completed cancer treatment at the time of the survey into the off-treatment group, based on the answer to the question about treatment status. |
| Bias | 9 | Describe any efforts to address potential sources of bias | N/A | We could not handle the selection bias because this study used convenient sampling through the commercial cancer panel system of Macromill, Inc. However, we discussed the limitation of our study and interpreted our results under the study limitation. |
| Study size | 10 | Explain how the study size was arrived at | N/A | This study was conducted for all cancer survivors who had registered with the commercial cancer panel system of Macromill, Inc. |

Continued on next page

| Quantitative variables | 11 | Explain how quantitative variables were handled in the analyses. If applicable, describe which groupings were chosen and why | 9-13 | Please see the ‘Statistical analyses’ in the Methods and materials. |
| --- | --- | --- | --- | --- |
| Statistical methods | 12 | (*a*) Describe all statistical methods, including those used to control for confounding | 9-13 | Please see the ‘Statistical analyses’ in the Methods and materials. |
|  |  | (*b*) Describe any methods used to examine subgroups and interactions | 9-13 | Example: Thereafter, we compared the mean scores between the on-treatment and off-treatment groups using Welch’s t-test. |
|  |  | (*c*) Explain how missing data were addressed | 6-9, 13-14 | We excluded Fifty-five participants whose treatment status at the time of the survey was unknown. Other participants’ characteristics was not missed (Please see Table 1). Some items of the Patient Activation Measure-13 was missed, but patient activation score could be calculated for all participants (Please see Table 2). |
|  |  | (*d*) *Cohort study*—If applicable, explain how loss to follow-up was addressed  *Case-control study*—If applicable, explain how matching of cases and controls was addressed  *Cross-sectional study*—If applicable, describe analytical methods taking account of sampling strategy | N/A |  |
|  |  | (*e*) Describe any sensitivity analyses | N/A |  |
| Results | | | | |
| Participants | 13* | (a) Report numbers of individuals at each stage of study—eg numbers potentially eligible, examined for eligibility, confirmed eligible, included in the study, completing follow-up, and analysed | 6-7 | Of the 693 participants who returned the online questionnaire, 138 were excluded who were diagnosed with cancer when they were younger than 20, and 55 whose treatment status at the time of the survey was unknown. Finally, data from a total of 500 YA cancer survivors were analyzed for this study. |
|  |  | (b) Give reasons for non-participation at each stage | 6-7 | Of the 693 participants who returned the online questionnaire, 138 were excluded who were diagnosed with cancer when they were younger than 20, and 55 whose treatment status at the time of the survey was unknown. Finally, data from a total of 500 YA cancer survivors were analyzed for this study. |
|  |  | (c) Consider use of a flow diagram | N/A | We judged that numbers of individuals at each stage of study could be adequately explained without the use of a flow of diagram. |
| Descriptive data | 14* | (a) Give characteristics of study participants (eg demographic, clinical, social) and information on exposures and potential confounders | 13-15 | Please see Table 1. |
|  |  | (b) Indicate number of participants with missing data for each variable of interest | 13-16 | Participants’ characteristics was not missed (Please see Table 1). Some items of the Patient Activation Measure-13 was missed, but patient activation score could be calculated for all participants (Please see Table 2). |
|  |  | (c) *Cohort study*—Summarise follow-up time (eg, average and total amount) | N/A |  |
| Outcome data | 15* | *Cohort study*—Report numbers of outcome events or summary measures over time | N/A |  |
|  |  | *Case-control study—*Report numbers in each exposure category, or summary measures of exposure | N/A |  |
|  |  | *Cross-sectional study—*Report numbers of outcome events or summary measures | 15-16 | Please see Table 2. |
| Main results | 16 | (*a*) Give unadjusted estimates and, if applicable, confounder-adjusted estimates and their precision (eg, 95% confidence interval). Make clear which confounders were adjusted for and why they were included | N/A | This study confirmed the psychometric properties and measurement invariance of the Patient Activation Measure-13 under the differences on characteristics between young adult cancer survivors during and after treatment. |
|  |  | (*b*) Report category boundaries when continuous variables were categorized | N/A | We did not categorized continuous variables. |
|  |  | (*c*) If relevant, consider translating estimates of relative risk into absolute risk for a meaningful time period | N/A | We did not used the relative and absolute risk. |

Continued on next page

| Other analyses | 17 | Report other analyses done—eg analyses of subgroups and interactions, and sensitivity analyses | 13-20 | We examined the psychometric properties and measurement invariance separately for young adult cancer survivors |
| --- | --- | --- | --- | --- |
| Discussion | | | | |
| Key results | 18 | Summarise key results with reference to study objectives | 20 | Example: Our study demonstrated that the feasibility, reliability, and validity of the Japanese version of the PAM-13 were confirmed… |
| Limitations | 19 | Discuss limitations of the study, taking into account sources of potential bias or imprecision. Discuss both direction and magnitude of any potential bias | 24 | Example: Most of our study participants were women, reflecting the high proportion of female cancer survivors aged 20–39 years in Japan… |
| Interpretation | 20 | Give a cautious overall interpretation of results considering objectives, limitations, multiplicity of analyses, results from similar studies, and other relevant evidence | 20-24 | Please see the Discussion. |
| Generalisability | 21 | Discuss the generalisability (external validity) of the study results | 24 | Example: Most of our study participants were women, reflecting the high proportion of female cancer survivors aged 20–39 years in Japan. Thus, the results may not be generalizable to Japanese male survivors. |
| Other information | |  | | |
| Funding | 22 | Give the source of funding and the role of the funders for the present study and, if applicable, for the original study on which the present article is based | Submission system | Please see the Financial Disclosure. |

*Give information separately for cases and controls in case-control studies and, if applicable, for exposed and unexposed groups in cohort and cross-sectional studies.

**Note:** An Explanation and Elaboration article discusses each checklist item and gives methodological background and published examples of transparent reporting. The STROBE checklist is best used in conjunction with this article (freely available on the Web sites of PLoS Medicine at http://www.plosmedicine.org/, Annals of Internal Medicine at http://www.annals.org/, and Epidemiology at http://www.epidem.com/). Information on the STROBE Initiative is available at www.strobe-statement.org.
