# Supplementary material for: Adaptation and measurement invariance of the 13-item version of Patient Activation Measure across Japanese young adult cancer survivors during and after treatment: A cross-sectional observational study
Source: PLoS One. 2023 Sep 19;18(9):e0291821. doi: 10.1371/journal.pone.0291821 (PMC10508623; doi:10.1371/journal.pone.0291821)
Supplement: S1 Table — (DOCX) [file pone.0291821.s002.docx]

**S1 Table. Items on the PAM-13.**

| Item 1 | When all is said and done, I am the person who is responsible for taking care of  my health. |
| --- | --- |
| Item 2 | Taking an active role in my own health care is the most important thing that  affects my health. |
| Item 3 | I am confident I can help prevent or reduce problems associated with my health. |
| Item 4 | I know what each of my prescribed medications do. |
| Item 5 | I am confident that I can tell whether I need to go to the doctor or whether I can  take care of a health problem myself. |
| Item 6 | I am confident that I can tell a doctor concerns I have even when he or she  does not ask. |
| Item 7 | I am confident that I can follow through on medical treatments I may need to  do at home. |
| Item 8 | I understand my health problems and what causes them. |
| Item 9 | I know what treatments are available for my health problems. |
| Item 10 | I have been able to maintain (keep up with) lifestyle changes, like eating right  or exercising. |
| Item 11 | I know how to prevent problems with my health. |
| Item 12 | I am confident I can figure out solutions when new problems arise with my health. |
| Item 13 | I am confident that I can maintain lifestyle changes, like eating right and  exercising, even during times of stress. |
| Response Options: Disagree Strongly (1), Disagree (2), Agree (3), Agree Strongly (4), and Not Applicable. | |
